# The NAMPT–MMP-9 Axis Contributes to Chronic Inflammation in Cystic Fibrosis

**DOI:** 10.3390/ijms27219496

**Published:** 2026-10-24

**Authors:** Laura Lori, Irene Fiorilla, Beatrice Ghezzi, Marco Pedrazzi, Alberto Maria Todesco, Francesca Mazzola, Roberta De Tullio, Federico Cresta, Carlo Castellani, Mauro Patrone, Nadia Raffaelli, Valentina Audrito, Monica Averna

**Affiliations:** 1 Department of Experimental Medicine, University of Genoa, 16132 Genoa, Italy; laura.lori@edu.unige.it (L.L.); marco.pedrazzi@unige.it (M.P.); detullio@unige.it (R.D.T.); 2 Department of Science and Technological Innovation, University of Eastern Piedmont, 15121 Alessandria, Italy; irene.fiorilla@uniupo.it (I.F.); beatrice.ghezzi@uniupo.it (B.G.); albertomaria.todesco@uniupo.it (A.M.T.); mauro.patrone@uniupo.it (M.P.); 3 Department of Clinical Sciences, Polytechnic University of Marche, 60126 Ancona, Italy; f.mazzola@staff.univpm.it; 4 Cystic Fibrosis Center, IRCCS Istituto Giannina Gaslini, 16147 Genoa, Italy; federicocresta@gaslini.org (F.C.); carlocastellani@gaslini.org (C.C.); 5 Research Laboratories, Research and Innovation Department (DAIRI), IRCCS AOU SS Antonio e Biagio e Cesare Arrigo, 15121 Alessandria, Italy; 6 Department of Agriculture, Food and Environmental Sciences, Polytechnic University of Marche, 60131 Ancona, Italy; n.raffaelli@staff.univpm.it

**Keywords:** cystic fibrosis, NAMPT, eNAMPT, MMP-9, ERK1/2, inflammation

## Abstract

Chronic inflammation is a major driver of lung damage in cystic fibrosis (CF), yet the molecular mechanisms that sustain this self-reinforcing inflammatory state remain incompletely understood. Nicotinamide phosphoribosyltransferase (NAMPT), the rate-limiting enzyme in NAD biosynthesis, can also act extracellularly (eNAMPT) as a pro-inflammatory mediator. Here, we investigated the relationship between NAMPT and matrix metalloproteinase-9 (MMP-9) in CF. Peripheral blood mononuclear cells (PBMCs) from people with CF (pwCF) displayed increased intracellular NAMPT and MMP-9 expression, together with enhanced AKT and ERK1/2 phosphorylation, compared with non-CF controls. Circulating eNAMPT levels were elevated in pwCF and positively correlated with MMP-9 levels. Although baseline eNAMPT levels were not significantly associated with FEV1% predicted, in a small longitudinal cohort, eNAMPT decreased following one year of elexacaftor/tezacaftor/ivacaftor (ETI) treatment, while FEV1% predicted increased in six of seven patients with paired measurements. In vitro, recombinant NAMPT increased MMP-9 expression and enzymatic activity in PBMCs from healthy donors. Pharmacological treatment with FK866 reduced endogenous eNAMPT release and attenuated NAMPT-induced MMP-9 production. Mechanistically, eNAMPT triggered rapid AKT activation followed by sustained ERK1/2 phosphorylation. Pharmacological inhibition of the MEK/ERK pathway attenuated NAMPT-induced MMP-9 activity, supporting a functional contribution of ERK1/2 signaling to NAMPT-mediated MMP-9 activation. Together, these findings identify a functional NAMPT–MMP-9 axis associated with inflammatory signaling in CF. Further studies are needed to establish its contribution to pulmonary inflammation and to determine the clinical significance of circulating eNAMPT in larger patient cohorts.

## 1. Introduction

Cystic Fibrosis (CF) is an autosomal recessive disease caused by mutations in the cystic fibrosis transmembrane conductance regulator (CFTR) gene, which impair the expression or function of the CFTR protein leading to a multisystemic disease characterized by chronic airway infection and progressive respiratory insufficiency [1]. One of the most troubling aspects of this condition is the chronic and abnormal inflammation that manifests in patients, significantly contributing to disease progression and associated complications.

The introduction of CFTR modulators [2] and, particularly Elexacaftor/Tezacaftor/Ivacaftor (ETI) into clinical practice, demonstrated significant benefits for the majority of people with CF (pwCF), improving lung function and quality of life [3]. Moreover, it has also been reported that ETI could have anti-inflammatory properties [4], reducing several lung and serum pro-inflammatory cytokines as well as inflammatory neutrophil and macrophage phenotypes [5,6,7]. Unfortunately, a subset of patients affected by CF still do not have an effective modulator treatment, either because they carry mutations which preclude CFTR protein production or because, despite being potentially treatable, do not respond adequately to current molecular therapies. These patients suffer from persistent inflammation, particularly in the respiratory tract, contributing to disease progression and deterioration in quality of life.

The relationship between CF and severe airway inflammation is not fully understood due to the complexity and the heterogeneity of the inflammatory response, which involves molecular and cellular mechanisms that are still unclear.

One inflammatory mediator of increasing interest is matrix metalloprotease-9 (MMP-9), which reflects airway inflammation in CF progression through its multifaceted role in extracellular matrix degradation, immune modulation, and amplification of neutrophilic inflammation [8,9,10]. Elevated MMP-9 levels are consistently found in the sputum, bronchoalveolar lavage fluid, and plasma of pwCF, correlating inversely with lung function parameters such as forced expiratory volume in 1 s (FEV1), indicating more severe disease [11]. Based on RNA-Seq evaluation of whole blood gene expression changes in response to lumacaftor/ivacaftor, it has been reported that MMP-9 expression was reduced in therapy responder patients [12]. Moreover, in our previous studies we have demonstrated that effective CF therapies, including CFTR modulators like Ivacaftor and ETI, downregulate plasmatic MMP-9 levels in positive clinical responders, suggesting that MMP-9 reduction reflects therapeutic efficacy and disease amelioration [13]. These findings highlight the importance of MMP-9 in inflammatory processes and tissue remodeling in CF suggesting that targeted interventions to modulate MMP-9 activity could represent a promising therapeutic strategy to mitigate inflammation and slow disease progression.

Induction of MMPs activity was previously associated with the pro-inflammatory and pro-invasive features of the enzyme/cytokine nicotinamide phosphoribosyltransferase (NAMPT) in macrophages and tumor models [14,15,16]. NAMPT, the key-limiting enzyme controlling NAD biosynthesis from nicotinamide, is an emerging novel damage-associated molecular protein (DAMP) [17]. In fact, it can be released in the extracellular space, where it behaves as a mediator of inflammation through binding to cell membrane receptors, including toll-like receptor 4 (TLR4) or C-C chemokine receptor 5 (CCR5) [18]. Extracellular NAMPT (eNAMPT) acts as a cytokine-like protein modulating the immune response, triggering intracellular signaling that promotes differentiation/polarization of myeloid cells, activation of inflammasome and secretion of inflammatory cytokines [18,19]. Several studies have reported elevated levels of eNAMPT in various metabolic and inflammatory conditions such as diabetes, obesity, atherosclerosis, autoimmune diseases, inflammatory bowel disease, lung injury and cancer [19]. In a paper published in 2011, the NAMPT gene emerged as one of the top 50 up-regulated genes in CF versus non-CF bronchial epithelium in a global gene expression analysis [20]. More recently, the NAMPT gene was found among the top 10 up-regulated genes in LPS activated CF neutrophils as compared to controls [21].

However, the functional role of NAMPT and its relationship with MMP-9 have not yet been investigated in the context of CF. The aim of this study is to address this knowledge gap by exploring the potential interplay between NAMPT and MMP-9 in amplifying the inflammatory response in pwCF, with a focus on their functional interactions and the underlying signaling pathways.

## 2. Results

### 2.1. MMP-9, NAMPT and Pro-Inflammatory Signaling Are Sustained in pwCF Compared to Non-CF Normal Subjects

**Figure 1:**
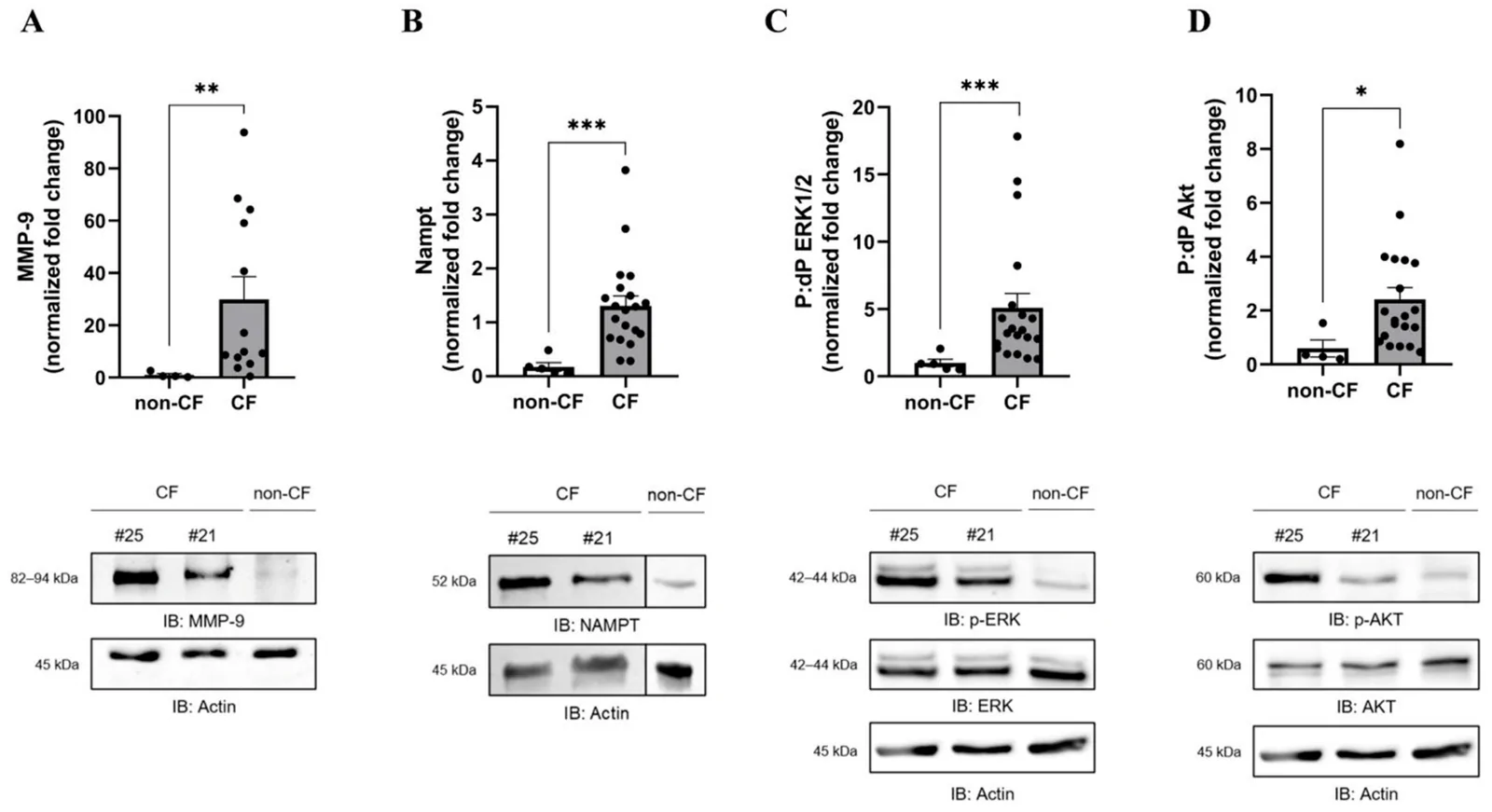
**Intracellular protein levels of MMP-9, NAMPT and pro-inflammatory signaling molecules in PBMCs from pwCF and healthy donors.** PBMC lysates from healthy donors (non-CF; (**A**,**D**): *n* = 4 and (**B**,**C**): *n* = 5) and pwCF (CF) were analyzed by Western blot for (**A**) MMP-9 protein levels (*n* = 13), (**B**) NAMPT protein levels (*n* = 21), (**C**) phosphorylated ERK1/2 (p-ERK1/2) normalized to total ERK1/2 (*n* = 20), and (**D**) phosphorylated AKT (p-AKT) normalized to total AKT (*n* = 20). β-actin was used to normalize the quantified protein levels in (**A**,**B**) and as loading control in (**C**,**D**). Representative immunoblots are shown below each graph. Data are expressed as fold change relative to non-CF donors (*n* = 5). Bars represent mean ± SEM. Statistical significance was determined using Mann–Whitney test: * *p*  ≤  0.05, ** *p*  ≤  0.01, *** *p*  ≤  0.001.

First, we analyzed the expression levels of MMP-9, NAMPT and pro-inflammatory signaling pathways in PBMC derived from the same cohort of pwCF vs. non-CF subjects. As indicated in Figure 1A, the MMP-9 intracellular levels of CF PBMCs are statistically significantly higher than those measured in non-CF PBMC, confirming our previous evidence [13]. To our knowledge NAMPT was revealed as highly expressed genes in CF bronchial epithelium [20]. Here, we measured intracellular protein expression of NAMPT in PBMC derived from pwCF vs. non-CF, as reported in Figure 1B, highlighting, as for MMP-9, a strong expression in PMBC from pwCF. These results suggest that the two proteins not only could be markers of the pathology but also involved in supporting inflammatory conditions in CF. To further investigate this hypothesis, we firstly evaluated the signaling pathways possibly involved in sustaining the inflammation in CF. Particularly, we investigated on the activation of AKT and ERK1/2 signaling, known to be involved not only in inflammation but also in the up-regulation of MMP-9 and NAMPT [17,22]. Data reported that the phosphorylation levels of ERK1/2 and AKT are significantly increased in PBMCs of pwCF in comparison to those measured in non-CF (Figure 1C,D), indicating that these signaling pathways are activated in the same PBMCs from pwCF expressing significantly high levels of both NAMPT and MMP-9.

### 2.2. Circulating eNAMPT Levels Are Elevated in pwCF and Correlate with MMP-9

**Figure 2:**
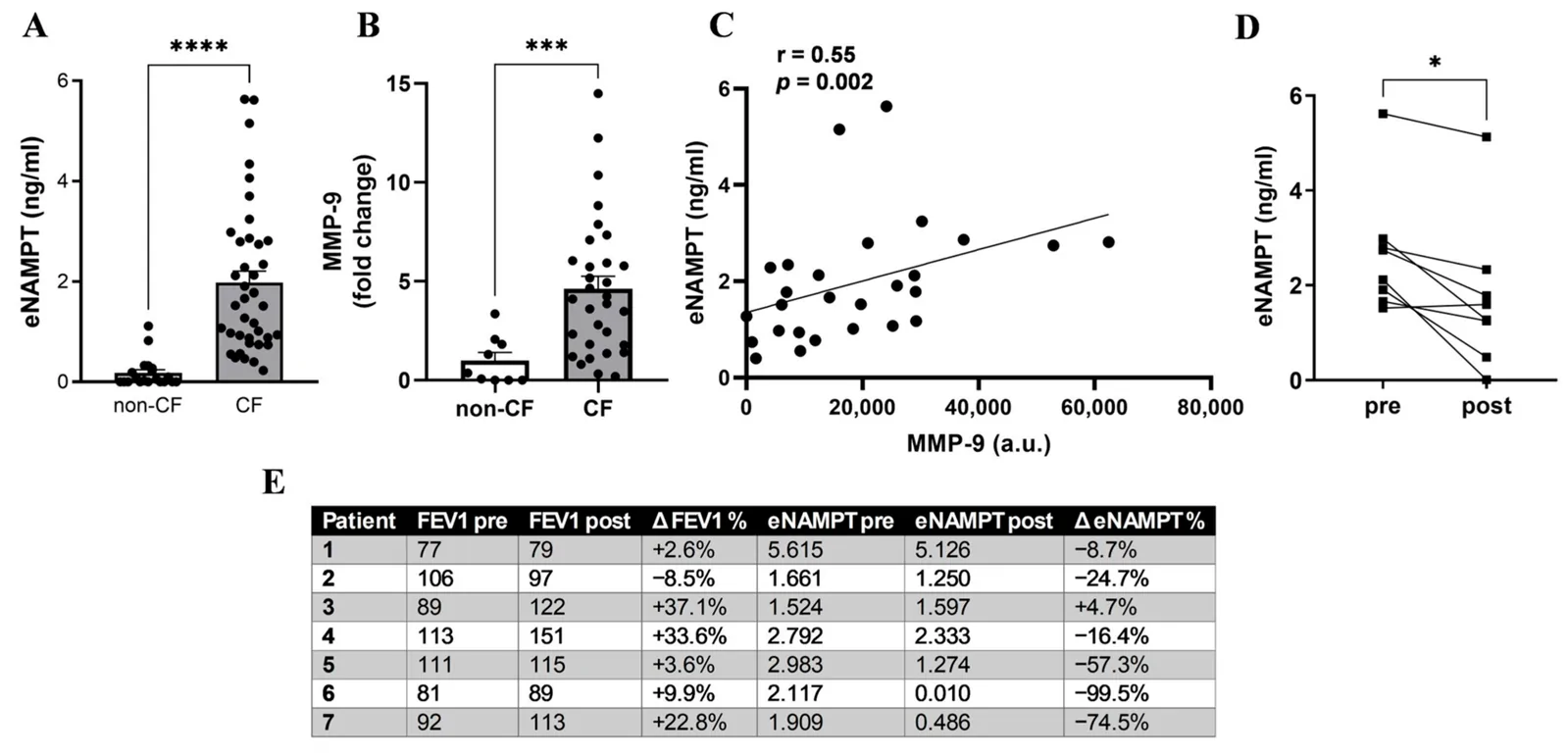
**Quantification and correlation analysis of MMP-9 and NAMPT plasma levels in pwCF and healthy donors.** Plasma samples from healthy donors (non-CF) and pwF (CF) were analyzed by ELISA to determine eNAMPT concentration (**A**) in CF (*n* = 40) compared to non-CF subjects (*n* = 20) and (**D**) in paired samples collected before (pre) and after (post) ETI treatment (*n* = 8). (**B**) Plasma MMP-9 levels were determined by Western blot analysis. (**C**) Correlation analysis between plasma eNAMPT concentration and MMP-9 protein levels was performed using Spearman’s rank correlation test. Data are shown as mean ± SEM or as individual paired values. Statistical significance was determined using Mann–Whitney test (**A**,**B**) and Wilcoxon test in (**D**). * *p*  ≤  0.05, *** *p*  ≤  0.001, **** *p*  ≤  0.0001. (**E**) Table reporting the values and the differences (delta value) between eNAMPT levels (ng/mL) and % FEV1 in 7 CF patients pre- post-ETI treatment.

We previously identified increased NAMPT expression in PBMCs from pwCF. Moreover, the NAMPT gene was reported among the top 50 upregulated genes in CF bronchial epithelium compared with non-CF controls [20]. We therefore investigated whether NAMPT could also be detected as a circulating protein (eNAMPT) in pwCF and whether its levels were associated with MMP-9. ELISA analysis revealed significantly higher plasma eNAMPT levels in pwCF (*n* = 40) than in age-comparable non-CF controls (*n* = 20), with an approximately 11.2-fold increase (Figure 2A). MMP-9 protein levels were also significantly elevated in the same patient cohort (Figure 2B), consistent with our previous findings [13]. Furthermore, circulating eNAMPT levels positively correlated with MMP-9 levels in pwCF (*n* = 27; Spearman’s r = 0.55, *p* = 0.002; Figure 2C).

We next investigated changes in circulating eNAMPT following one year of elexacaftor/tezacaftor/ivacaftor (ETI) treatment. In the available longitudinal cohort of eight patients, eNAMPT levels significantly decreased following treatment (Figure 2D; *p* = 0.031), paralleling the effect on MMP-9 levels [13]. To further investigate the clinical relevance of circulating eNAMPT, we analyzed its relationship with lung function at baseline and longitudinally following ETI (FEV1%, Table S1). At baseline, no significant correlation was observed between plasma eNAMPT levels and FEV1% predicted (Spearman’s r = −0.075, *p* = 0.67, *n* = 35). We then examined the seven patients for whom paired pre- and post-ETI measurements of both eNAMPT and FEV1 were available. In six out of seven patients, eNAMPT levels decreased following ETI, whereas FEV1% predicted increased (Figure 2E). Moreover, changes in eNAMPT levels were not significantly correlated with changes in FEV1% predicted (Spearman’s r = 0.14, *p* = 0.76, *n* = 7). Overall, these findings indicate that circulating eNAMPT levels are elevated in pwCF, correlate with MMP-9 levels, and decrease following ETI treatment. However, the available data do not establish a direct relationship between circulating eNAMPT and lung function. Further studies in larger longitudinal cohorts are required to clarify the clinical significance of these observations.

### 2.3. eNAMPT Increases MMP-9 Expression and Activity, Reduced by NAMPT Inhibition

**Figure 3:**
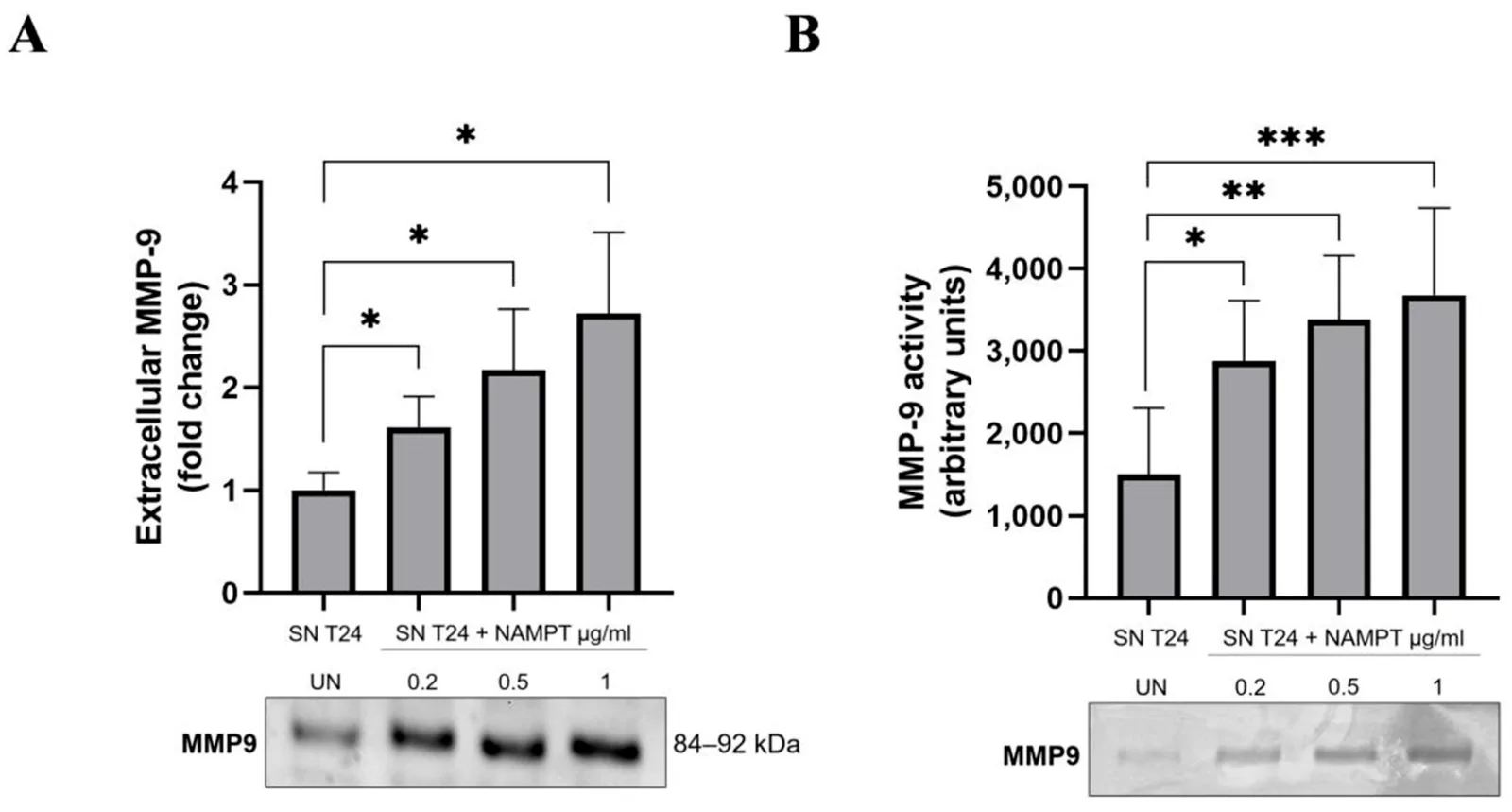
**Extracellular MMP-9 expression and activity in conditioned media from healthy donor PBMCs following rNAMPT treatment.** Healthy donor PBMCs were treated with increasing concentrations of recombinant (r)NAMPT (0.2–0.5–1 μg/mL). After 24 h, conditioned media were collected and analyzed by (**A**) Western blot to determine extracellular MMP-9 expression (*n* = 4) and (**B**) gelatin zymography to evaluate MMP-9 enzymatic activity (*n* = 8). A representative immunoblots and zymogram are shown below each graph. Data were normalized to Ponceau Red staining (Supplementary Figure S1A). Statistical significance was determined using one-way ANOVA (*p* = 0.0139 and *p* = 0.0002), followed by Dunnett’s multiple comparisons test: * *p*  ≤ 0.05, ** *p*  ≤ 0.01, *** *p*  ≤ 0.001.

To investigate the functional relationship between NAMPT and MMP-9, PBMCs isolated from healthy donors were cultured for 24 h in the presence of increasing concentrations of recombinant human NAMPT (rNAMPT). As shown in Figure 3, rNAMPT treatment significantly increased both MMP-9 protein levels (Figure 3A) and enzymatic activity (Figure 3B) in the culture supernatants compared with untreated cells. Protein levels were normalized to total protein using Ponceau Red staining (Supplementary Figure S1A).

**Figure 4:**
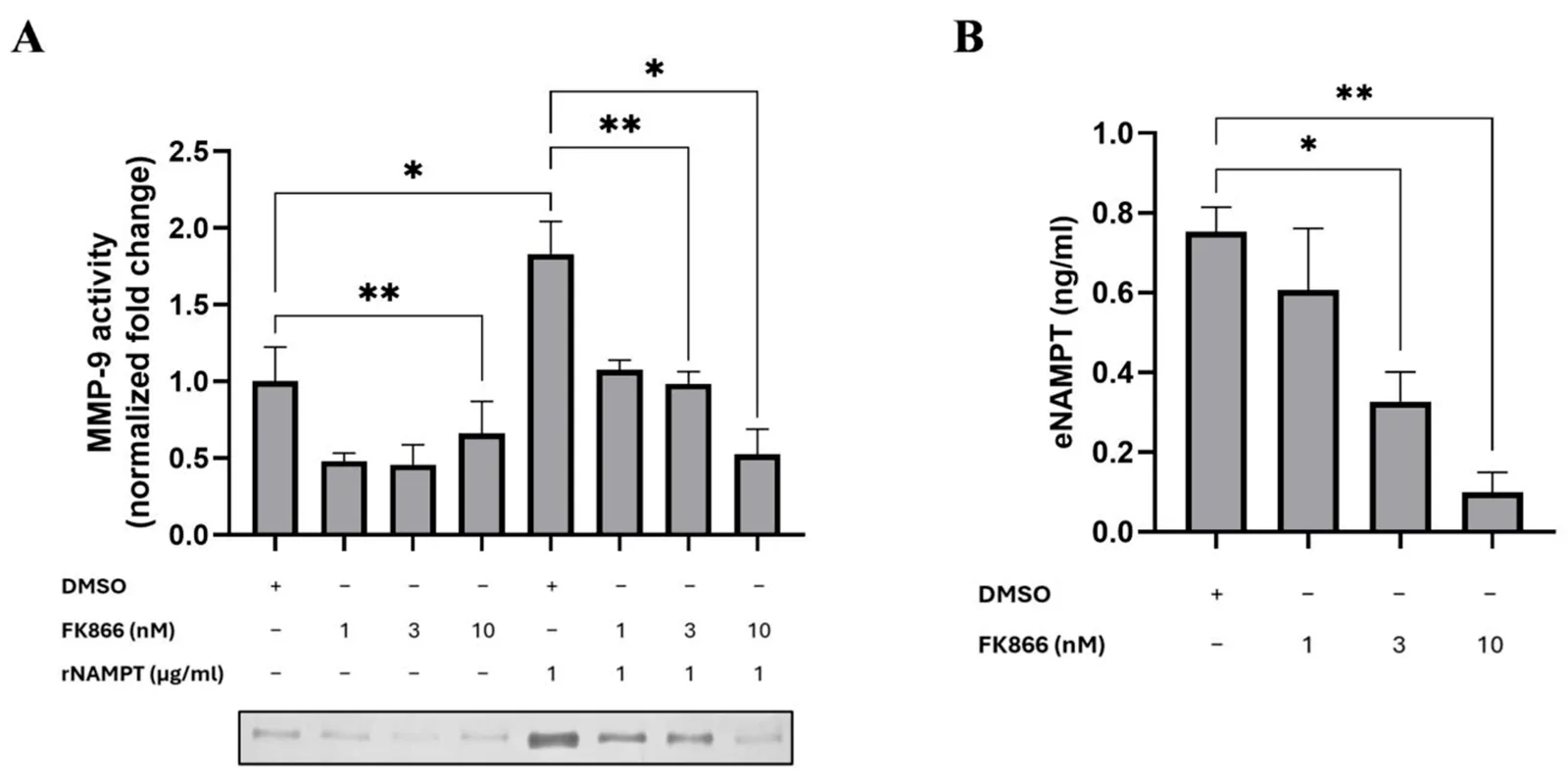
**Analysis of extracellular MMP-9 activity and eNAMPT secretion following rNAMPT stimulation and/or FK866 treatment.** Healthy donor PBMCs were treated with FK866 (1, 3 or 10 nM) in the absence or presence of rNAMPT (1 µg/mL). After 24 h, conditioned media were collected and analyzed by (**A**) gelatin zymography to assess MMP-9 enzymatic activity (*n* = 7, *n* = 4 for samples treated with FK866 1nM) and (**B**) ELISA assay to determine eNAMPT concentration in conditioned media from untreated and FK866-treated samples (*n* = 3). A representative zymogram is shown below the corresponding graph. Data in B were normalized to Ponceau Red staining (Supplementary Figure S1B). Bars represent mean ± SEM. Statistical significance was determined using mixed-effects analysis, followed by Dunnett’s multiple comparisons test for DMSO vs. all FK866 treatments (*p* = 0.03) and for DMSO + rNAMPT vs. rNAMPT + all FK866 treatments (*p* = 0.0045); DMSO vs. DMSO + rNAMPT treatments were evaluated by paired *t*-test. * *p* ≤ 0.05, ** *p* ≤ 0.01.

We next investigated the effects of FK866, a pharmacological inhibitor of NAMPT enzymatic activity, on MMP-9 activity in the presence or absence of exogenous rNAMPT. In the presence of rNAMPT, FK866 treatment reduced MMP-9 activity under the experimental conditions tested (Figure 4A). This effect is unlikely to result from inhibition of the enzymatic activity of extracellular rNAMPT, since PRPP and ATP, which are required to sustain its catalytic activity, are either absent or present at low concentrations. However, we cannot exclude the possibility that FK866 binding to rNAMPT may interfere with its extracellular signaling function. Importantly, FK866 also showed a trend toward reducing basal MMP-9 activity in the supernatants of PBMCs cultured without exogenous rNAMPT, although statistical significance was not reached across all tested concentrations (Figure 4A). These findings suggest that FK866 can modulate the NAMPT–MMP-9 axis in PBMCs, potentially through effects on both extracellular NAMPT signaling and endogenous NAMPT-related mechanisms. The present experiments, however, do not distinguish between these possibilities.

Importantly, the low concentrations of FK866 used in this study (1–10 nM) do not affect PBMC viability after 24 h of treatment, whereas reduced cell viability has been reported only after 72 h at similar concentrations [23].

We also examined extracellular NAMPT levels in conditioned media following FK866 treatment (Figure 4B). FK866 treatment significantly reduced endogenous eNAMPT levels in the culture supernatants without affecting total protein content (Supplementary Figure S1B). Although these findings suggest that NAMPT inhibition may affect eNAMPT release, the limited number of independent samples (*n* = 3) warrants caution and highlights the need for further validation.

Collectively, these experiments support a functional relationship between NAMPT and MMP-9 in PBMCs. Further studies are required to clarify the mechanisms underlying the effects of FK866 and to distinguish the respective contributions of intracellular and extracellular NAMPT.

### 2.4. Recombinant NAMPT Activates AKT and ERK1/2 Signaling, and ERK1/2 Inhibition Attenuates MMP-9 Activity

**Figure 5:**
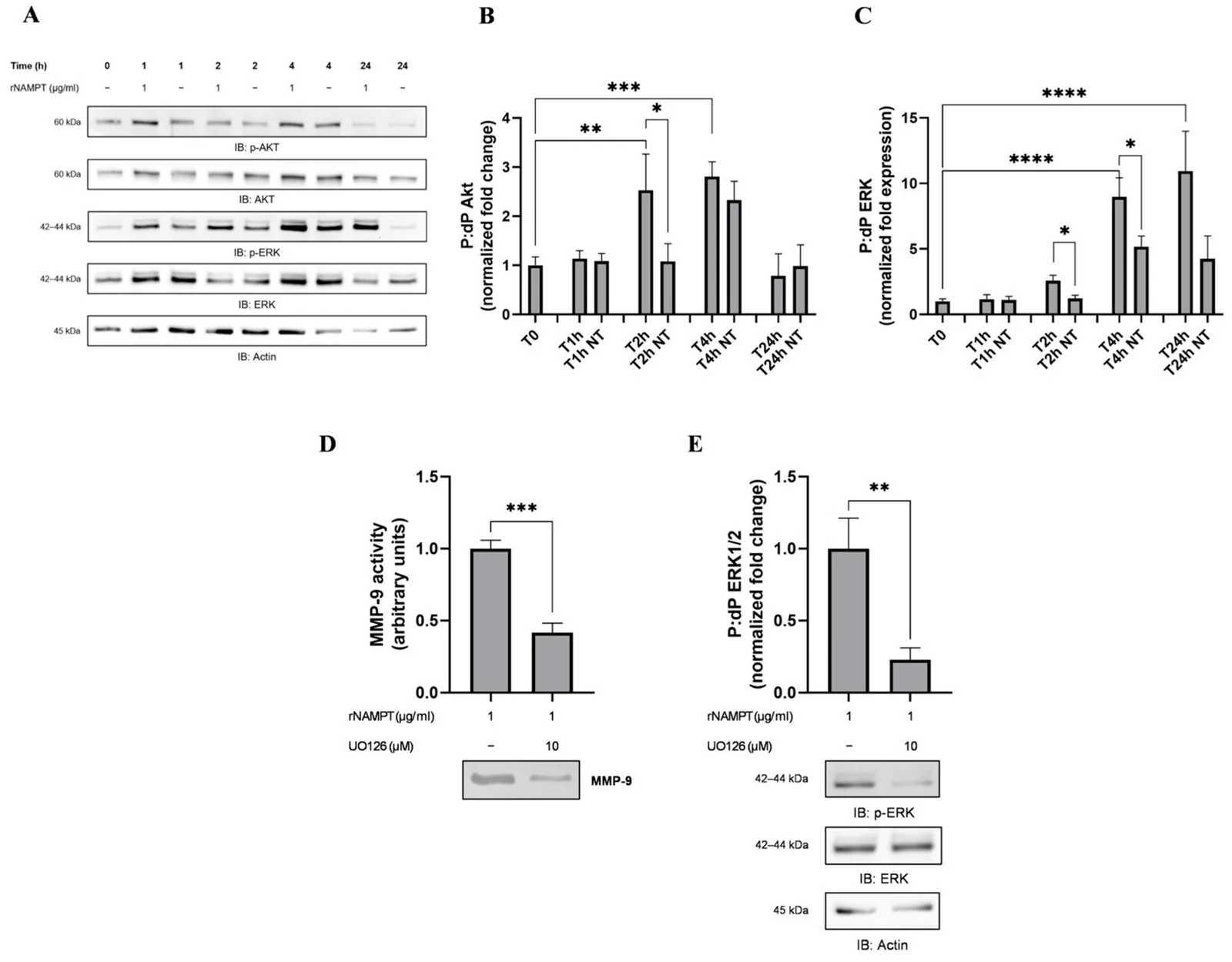
**Intracellular Akt and ERK1/2 phosphorylation following rNAMPT stimulation and U0126-mediated inhibition of ERK1/2 phosphorylation**. Healthy donor PBMCs were treated with rNAMPT (1 µg/mL) for 1, 2, 4 and 24 h. Cell lysates were analyzed by Western blot; representative immunoblots are shown for each protein (**A**), to determine (**B**) Akt (*n* = 11 for 1 h and 1 h NT; *n* = 10 for T0, 4 h and 4 h NT; *n* = 8 for 2 h; *n* = 5 for 2 h NT, 24 h and 24 h NT) and (**C**) ERK1/2 phosphorylation levels (*n* = 11 for T0, 1 h and 1 h NT; *n* = 10 for 2 h; *n* = 8 for 4 h and 4 h NT; *n* = 6 for 2 h NT and 24 h NT). Phosphorylated proteins were normalized to their respective total proteins. β-actin was used as loading control. (**D**,**E**) Healthy donor PBMCs were treated with rNAMPT with or without U0126 for 24 h. Cell supernatants were analyzed by gelatin zymography for MMP-9 activity (**D**), while cell lysates were analyzed by Western blot for ERK1/2 phosphorylation levels (**E**). Bars represent mean ± SEM. (**B**) Statistical significance was determined using one-way ANOVA for T0, T1 h, T2 h, T4 h, and T24 h (*p* = 0.0002), followed by Dunnett’s multiple comparisons test; the evaluation of the differences between treated and NT conditions (2 h and 4 h) was made using Mann–Whitney test (2 h) and Wilcoxon test (4 h). (**C**) Statistical significance was determined using one-way ANOVA for T0, T1 h, T2 h, T4 h, and T24 h (*p* < 0.0001), followed by Dunnett’s multiple comparisons test.; the evaluation of the differences between treated and NT conditions was made using unpaired *t*-test. (**D**,**E**) Statistical significance was determined by a paired *t*-test. * *p* ≤ 0.05, ** *p*  ≤ 0.01, *** *p*  ≤ 0.001, **** *p*  ≤ 0.0001.

In the last part of the study, we investigated the intracellular inflammatory signaling potentially involved to the activation of the NAMPT/MMP-9 axis. Since AKT and ERK1/2 phosphorylation was increased in PBMCs from pwCF (Figure 1), we examined their activation following rNAMPT stimulation in PBMCs from healthy donors. Phosphorylation levels of both AKT (Figure 5A,B) and ERK1/2 (Figure 5A,C) increased statistically significantly two hours after rNAMPT exposure. Then, we observed a divergent temporal profile between the two kinases. While AKT phosphorylation progressively declined over time (Figure 5B), ERK1/2 activation further increased, remaining sustained even after 24 h of rNAMPT stimulation (Figure 5C). Notably, at this late time point, the specificity of the rNAMPT-induced event appeared to be reduced.

To investigate whether ERK1/2 signaling contributes functionally to NAMPT-induced MMP-9 activity, we treated PBMCs with rNAMPT in the presence or absence of the MEK/ERK pathway inhibitor U0126. Pharmacological inhibition attenuated MMP-9 activity in the culture supernatant (Figure 5D) and reduced ERK1/2 phosphorylation (Figure 5E). These findings support a functional contribution of MEK/ERK signaling to the induction of MMP-9 activity following rNAMPT stimulation.

Taken together, these results indicate that rNAMPT activates AKT and ERK1/2 signaling in PBMCs and that pharmacological inhibition of the MEK/ERK pathway attenuates MMP-9 activity. Whether AKT signaling also contributes functionally to MMP-9 induction remains to be established.

## 3. Discussion

The chronic, non-resolving inflammatory response remains a major determinant of lung damage and progressive lung function decline in CF, even in the era of highly effective CFTR modulators. Although persistent neutrophilic inflammation and excessive protease activity are recognized hallmarks of CF lung disease [8,24], the mechanisms sustaining this inflammatory state remain incompletely understood. In the present study, we identify a functional relationship between eNAMPT and MMP-9 that may contribute to systemic inflammatory activation in pwCF. We found increased intracellular NAMPT and MMP-9 in PBMCs from pwCF, together with enhanced AKT and ERK1/2 phosphorylation. Circulating eNAMPT levels were also elevated and positively correlated with MMP-9 concentrations. In vitro experiments further showed that recombinant NAMPT stimulates MMP-9 expression and activity in PBMCs from healthy donors, supporting a functional link between these mediators.

Although CF is primarily considered a disease of the airway epithelium, immune dysregulation also involves circulating leukocytes [25,26,27,28]. PBMCs from pwCF display an inflammatory phenotype characterized by altered cytokine production and activation of MAPK- and PI3K-dependent signaling pathways [29,30]. Our findings extend this evidence by identifying NAMPT as a component of this phenotype. This observation is consistent with previous transcriptomic analyses reporting increased NAMPT expression in CF bronchial epithelial cells [20], suggesting that NAMPT dysregulation may involve both pulmonary and peripheral compartments. However, because our mechanistic experiments were primarily performed in PBMCs from healthy donors, further studies are needed to establish whether the same responses occur in immune cells from pwCF and within the CF lung microenvironment.

A major finding of this study is that recombinant NAMPT promotes MMP-9 expression and enzymatic activity in PBMCs. MMP-9 is a well-established mediator of CF lung pathology, where it contributes to extracellular matrix degradation and airway remodeling [22,31,32]. Increased MMP-9 levels have been detected in sputum, bronchoalveolar lavage fluid, and serum from pwCF, and have been associated with inflammatory activity and disease severity [33]. Our previous work also showed that circulating MMP-9 decreases in patients responding to ETI therapy [13]. The positive correlation between circulating eNAMPT and MMP-9 observed in the present study, together with the effects of recombinant NAMPT in vitro, suggests that eNAMPT may contribute to the regulation of MMP-9. Nevertheless, the clinical correlation is associative and does not establish a direct causal relationship in patients.

To investigate the mechanisms underlying this response, we examined AKT and ERK1/2 activation following recombinant NAMPT stimulation. The two pathways displayed distinct temporal profiles, with a more transient AKT response and sustained ERK1/2 phosphorylation. Importantly, pharmacological inhibition of MEK/ERK signaling with U0126 attenuated NAMPT-induced MMP-9 activity, supporting a functional contribution of this pathway. Whether AKT also contributes to NAMPT-induced MMP-9 regulation remains to be determined. These results identify ERK1/2 signaling as one mechanism involved in the cellular response to extracellular NAMPT, without excluding the participation of additional pathways.

We also found that FK866 reduced NAMPT-induced MMP-9 activity and showed a trend toward reducing basal MMP-9 activity in the absence of exogenous recombinant NAMPT. FK866 treatment was additionally associated with a reduction in endogenous eNAMPT levels in conditioned media, without affecting total protein content. These observations raise the possibility that intracellular NAMPT activity influences extracellular NAMPT release. However, the current experiments cannot distinguish between effects resulting from intracellular NAMPT inhibition and potential effects of FK866 on extracellular NAMPT signaling. The mechanisms controlling eNAMPT secretion therefore remain to be clarified. Previous studies have implicated post-translational modifications, including SIRT1-dependent deacetylation, in the regulation of NAMPT secretion [34]. Given the dependence of SIRT1 activity on NAD availability, a connection between intracellular NAD metabolism and eNAMPT release is conceivable, although this mechanism was not directly investigated here.

Another clinically relevant observation was the decrease in circulating eNAMPT following one year of ETI treatment. This finding is consistent with evidence that CFTR modulator therapy can attenuate systemic and airway inflammation [5,6,7] and parallels the previously reported reduction in circulating MMP-9 following ETI [13]. However, baseline eNAMPT levels were not significantly associated with FEV1% predicted. In the small longitudinal subset with paired measurements, eNAMPT decreased while FEV1% predicted increased in six of seven patients, but changes in the two parameters were not significantly correlated. These findings suggest that circulating eNAMPT may respond to CFTR modulator treatment, but larger longitudinal cohorts are required to establish its clinical significance and potential value as an inflammatory biomarker.

The potential relevance of the NAMPT–MMP-9 axis to tissue injury can also be considered in the broader context of infection-associated epithelial damage and remodeling. Studies of *Mycobacterium bovis* infection have described mechanisms regulating epithelial apoptosis and epithelial–mesenchymal transition (EMT), highlighting links between infectious stimuli and epithelial injury [35,36]. MMP-9 is a plausible mediator connecting inflammatory activation with extracellular matrix remodeling. However, these studies concern different experimental systems and do not establish a role for NAMPT in these processes. Moreover, our experiments did not directly assess airway epithelial responses, extracellular matrix degradation, or lung tissue remodeling. The involvement of the NAMPT–MMP-9 axis in these pulmonary processes therefore remains to be investigated.

Several limitations should be acknowledged. First, the longitudinal analysis following ETI treatment involved a small number of patients, limiting the interpretation of clinical associations. Second, the mechanistic experiments were mainly conducted in PBMCs from healthy donors and do not reproduce the cellular complexity of the CF lung microenvironment. Third, the cellular sources of circulating eNAMPT and MMP-9 were not determined. Finally, although our results support a functional relationship between NAMPT and MMP-9 and implicate MEK/ERK signaling in the response to extracellular NAMPT, the receptors and molecular mechanisms involved remain incompletely characterized. Further studies in disease-relevant models and respiratory samples will be needed to clarify the contribution of this axis to CF-associated pulmonary inflammation.

## 4. Materials and Methods

### 4.1. Patient and Healthy Donor (HD) Samples

All the participants gave their written informed consent prior to their inclusion in the study, including permission to store the samples and to use them for research. The study protocol conformed to the provisions of the Declaration of Helsinki and those of the G. Gaslini Children’s Hospital, Genoa, Italy. The Ethics Committee of Genoa approved the study under protocol number A-CF2014 460REG2014 (October 2014). Informed consent was obtained from all subjects involved in the study. Patient characteristics are reported in Table S1. Plasma samples of HDs were obtained through the local blood bank of the Alessandria Hospital (AOU-AL).

### 4.2. Peripheral Blood Mononuclear Cell (PBMC) Isolation and Plasma Collection

Prior to the processing, an aliquot of whole blood was collected and centrifuged twice (800× *g* for 10 min and 16,000× *g* for 10 min). The resulting plasma was then aliquoted and stored at −80 °C until required.

PBMCs from blood samples were isolated as described in [37] and then resuspended in culture medium (RPMI 1640, containing 10% (*v*/*v*) FBS, 10 U/mL penicillin, 100 µg/mL streptomycin, 2 mM L-glutamine) at 10^6^/mL cell density for subsequent analyses.

Blood samples were diluted with an equal volume of RPMI 1640 (Euroclone SpA, Milan, Italy) and carefully stratified over the Lympholyte^®^-H Cell Separation Media, following manufacturer’s instructions. Briefly, the stratified samples were centrifuged at 800× *g* for 20 min at 22 °C without brake. After centrifugation, PBMCs were collected at the interface between the upper layer, containing the plasma fraction, and the lower layer, containing Lympholyte^®^-H. In order to remove platelets, PBMCs were washed twice with RPMI 1640 and then resuspended in culture medium (RPMI 1640, containing 10% (*v*/*v*) FBS, 10 U/mL penicillin, 100 µg/mL streptomycin, 2 mM L-glutamine) at 10^6^/mL cell density.

### 4.3. eNAMPT Quantification by Enzyme-Linked Immunosorbent Assay (ELISA)

eNAMPT concentrations in HD and pwCF plasma and in supernatants from PBMC cultures were determined using a human NAMPT ELISA kit (Adipogen, Epalinges, Switzerland; AG-45A-0006YEK-KI01) according to the manufacturer’s instructions [38].

### 4.4. Western Blot Analysis

Freshly isolated CF and non-CF PBMCs were lysed at 107/mL via sonication in Laemmli sample buffer and heated for 5 min at 95 °C. Aliquots (30 μL) of each sample were loaded on 4–20% Mini- PROTEAN^®^ TGX™ Precast Protein Gels (Bio-Rad (Segrate, Milan, Italy), #4568084) for NAMPT detection [39] and alternatively on home-made SDS-PAGE (6% or 10%) for the other antigens, followed by transfer on nitrocellulose membranes (Bio-Rad Trans-Blot Turbo RTA Midi 0.2 µm Nitrocellulose Transfer Kit #1704271, and GE Healthcare, Chicago, IL, USA). For the detection of extracellular MMP-9 expression, plasma samples from pwCF (2 uL) were diluted in Laemmli sample buffer, heated for 5 min at 95 °C, and loaded onto home-made SDS-PAGE gels (6%).

Membranes were blocked 1 h at room temperature in 5% non-fat dry milk (SERVA Electrophoresis GmbH, #42590.01, Heidelberg, Germany) in Tris-buffer saline, 0.1% Tween20 (ITW Reagents S.R.L., #A4974,0500, Monza, MB, Italy) and incubated with the indicated antibodies: anti-NAMPT (1:2000, A300-779A, Bethyl Laboratories, Montgomery, TX, USA), anti-MMP-9 (1:1000, #3852), anti-P-ERK 1/2 (1:1000, #9101), anti-ERK 1/2 (1:1000, #9102), anti-P-Akt (1:1000, #9271) and anti-Akt (1:1000, #9272) all from Cell Signaling Technology (Danvers, MA, USA) and anti-β-actin (1:1000, sc-47778) from Santa Cruz Biotechnology (Dallas, TX, USA). Secondary reagents were goat anti-rabbit IgG HRP-conjugated (1:5000, BioRad Cat.#1706515; 1:5000, Cell Signaling Technology, #7074) and anti-mouse IgG HRP-conjugated (1:5000, Cell Signaling Technology, #7076).

Western blot chemiluminescence reactions were visualized with ECL (Clarity Western ECL Substrate, Bio-Rad, #170-5060, Amersham™ ECL Select™ Western Blotting Detection Reagent, GE Healthcare, RPN2235) acquired using iBright™ CL1500 Imaging System (Thermo Fisher Scientific,Waltham, MA, USA) or ChemiDoc™ XRS equipped with Quantity One Image Software 4.6.1 (Bio-Rad Laboratories). Densitometric analyses were performed using Bio-Rad Image Lab software 6.1 version. Protein expression for cell lysates was normalized to β-actin. Protein expression and activity for supernatant samples were normalized to total protein staining with Ponceau S. A stripping and re-probing procedure was applied to test the membranes with all the primary antibodies [13].

### 4.5. Preparation of Human NAMPT Recombinant Protein (rNAMPT)

Human recombinant NAMPT was obtained as described in [40]. Briefly, the pET15b plasmid harboring human NAMPT cDNA was transformed into ClearColi BL21 (DE3) cells (Lucigen) and protein expression was induced with 0.8 mM IPTG at 25 °C for 16 h. The recombinant protein was purified by Ni-NTA affinity chromatography followed by gel filtration chromatography on PD-10 column equilibrated and eluted in 50 mM HEPES pH 7.5, 0.2 M NaCl. To ensure removal of possible endotoxin contamination, the final preparation was passed through a Pierce High-Capacity Endotoxin Removal Spin Column equilibrated with endotoxin-free buffer consisting of 50 mM HEPES pH 7.5, 0.2 M NaCl. The LPS content of the final preparation was assessed using HEK-BLUE^TM^ hTLR4 reporter cells as described in [41]. Based on a standard curve generated with LPS, the preparation contained less than 3 pg LPS (0.0003 EU)/ug recombinant protein.

### 4.6. Treatment of PBMCs with rNAMPT

In order to trigger intracellular signaling pathways, PBMCs isolated from non-CF individuals were treated with recombinant human NAMPT at different concentrations, as indicated elsewhere, for 1, 2, 4, or 24 h. Where indicated, rNAMPT stimulation was performed in the presence or absence of the NAMPT inhibitor FK866 (Sigma-Aldrich,St. Louis, MO, USA) at 1, 3, or 10 nM. Alternatively, where indicated, rNAMPT stimulation was performed in presence or absence of MEK1/2 inhibitor U0126 (MedChemExpress, NJ, USA) at 10 μM. Briefly, PBMCs were seeded in 6-well plates at a 1 × 10^6^/mL cell density in complete RPMI 1640, with or without the stimulus. At the end of the stimulation period, cell suspensions were collected and centrifuged to separate cell pellets from supernatants. The obtained supernatants were aliquoted and either immediately frozen for subsequent zymography analyses or added to Laemmli sample buffer and subjected to heat inactivation for 5 min at 95 °C for Western blot analyses. Cell pellets were lysed via sonication in Laemmli sample buffer, heated for 5 min at 95 °C. All collected samples were stored at −80 °C until further analysis.

### 4.7. Zymography Analysis

Plasma samples or supernatants collected from PBMCs were diluted in a modified Laemmli sample buffer and subjected to zymography analyses as previously described [13]. The intensity of the MMP-9-dependent lytic bands was quantified by densitometric analysis using ImageJ 1.8.0_322 (NIH, USA) and normalized to total protein staining with Ponceau S.

### 4.8. Statistical Analysis

Statistical analyses were performed using GraphPad Prism version 9.0 (GraphPad Software Inc., La Jolla, CA, USA). Statistical significance was assessed using an unpaired or paired Student’s *t*-test, following a Shapiro–Wilk test to evaluate the normality of data distribution. Non-parametric Mann–Whitney or Wilcoxon test was also used where indicated. One-way ANOVA was also used where indicated. Correlations between variables were assessed using Spearman’s rank correlation test. Statistical significance was reported in the legends of each Figure.

Unless otherwise indicated, data in the Figures are presented as the mean  ±  SEM. For all statistical tests, the 0.05 level of confidence was accepted for statistical significance. Significance was represented as: * *p*  ≤  0.05, ** *p*  ≤  0.01, *** *p*  ≤  0.001, **** *p*  ≤  0.0001.

## 5. Conclusions

In conclusion, our study identifies a functional relationship between NAMPT/eNAMPT and MMP-9 in the context of cystic fibrosis (CF). We found increased intracellular NAMPT and MMP-9 levels in peripheral blood mononuclear cells (PBMCs) from people with CF (pwCF), elevated circulating eNAMPT levels that positively correlated with MMP-9, and a reduction in circulating eNAMPT following elexacaftor/tezacaftor/ivacaftor (ETI) treatment. In vitro experiments further showed that recombinant NAMPT increased MMP-9 expression and activity in PBMCs from healthy donors. Pharmacological inhibition with FK866 reduced NAMPT-induced MMP-9 activity and endogenous eNAMPT release, while also showing a trend toward reducing basal MMP-9 activity in the absence of exogenous rNAMPT. Moreover, inhibition of the MEK/ERK pathway attenuated NAMPT-induced MMP-9 activity, supporting a functional contribution of ERK1/2 signaling to this response.

**Figure 6:**
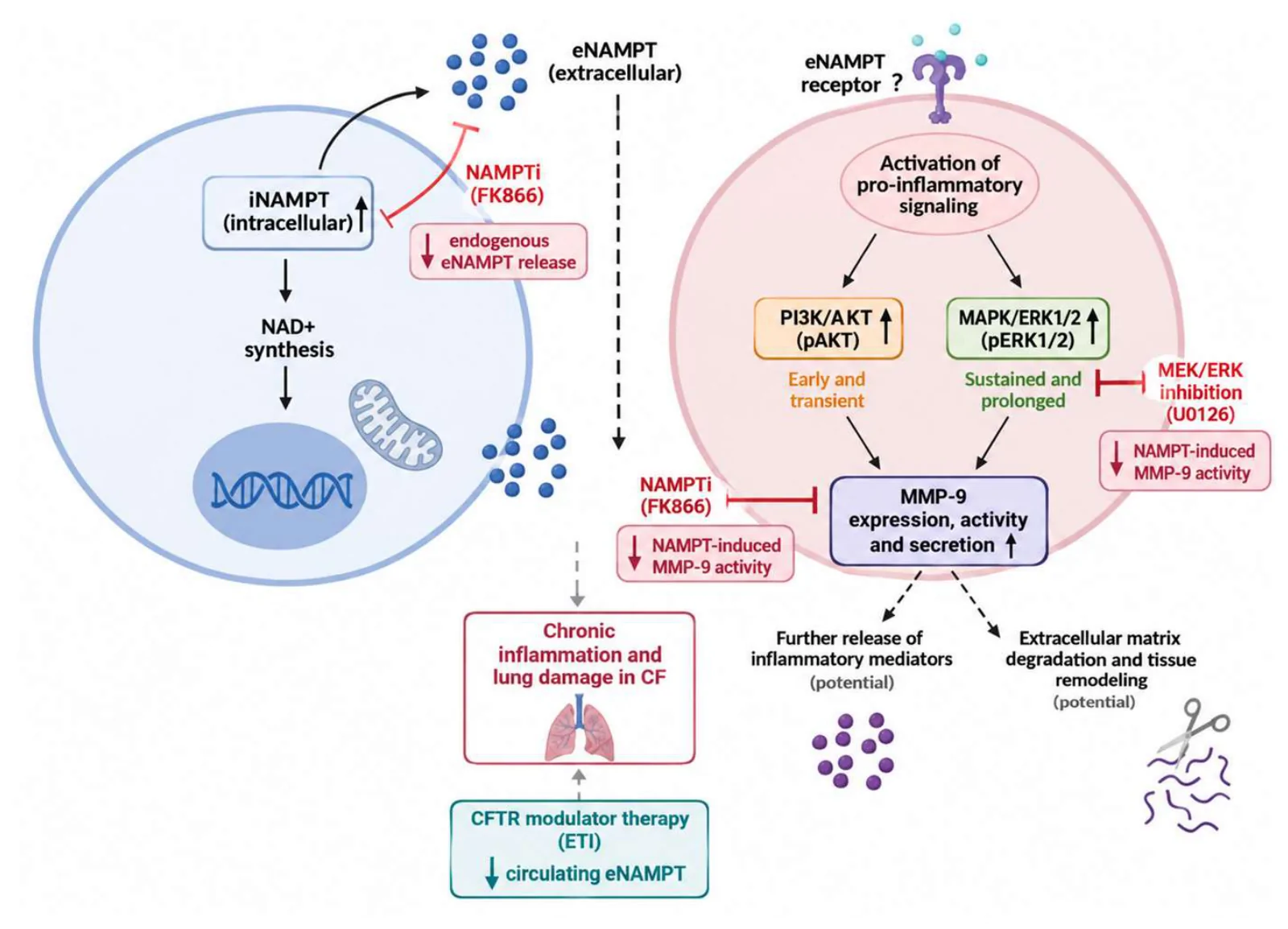
**Proposed model of the NAMPT–MMP-9 axis in cystic fibrosis (CF).** Intracellular NAMPT (iNAMPT) supports NAD^+^ biosynthesis and contributes to the release of extracellular NAMPT (eNAMPT). In PBMCs from people with CF (pwCF), increased intracellular NAMPT and circulating eNAMPT levels are associated with elevated MMP-9 levels. In vitro, recombinant NAMPT stimulates pro-inflammatory signaling in PBMCs from healthy donors, inducing rapid and transient AKT phosphorylation and sustained ERK1/2 phosphorylation, followed by increased MMP-9 expression, activity, and secretion. Pharmacological inhibition with FK866 reduces NAMPT-induced MMP-9 activity and endogenous eNAMPT release, whereas inhibition of the MEK/ERK pathway with U0126 attenuates NAMPT-induced MMP-9 activity. Circulating eNAMPT levels decrease following elexacaftor/tezacaftor/ivacaftor (ETI) treatment. The potential contribution of MMP-9 to further inflammatory mediator release, extracellular matrix degradation, and lung tissue remodeling remains to be established. Solid arrows indicate experimentally supported relationships, whereas dashed arrows represent proposed mechanisms that require further investigation. Inhibition is indicated by red bars.

Together, these findings support the involvement of a NAMPT–MMP-9 axis associated with inflammatory signaling in CF (Figure 6). However, the available data do not establish a direct relationship between circulating eNAMPT levels and lung function or demonstrate a causal role for this axis in pulmonary inflammation and tissue remodeling. Further studies in disease-relevant models and larger longitudinal patient cohorts are needed to clarify the mechanisms underlying NAMPT–MMP-9 interactions, establish their relevance to CF-associated lung inflammation, and determine whether circulating eNAMPT may have clinical biomarker potential.

## Data Availability

The data supporting the findings of this study are available from the corresponding authors upon reasonable request.

## Supplementary Materials

The following supporting information can be downloaded at: https://www.mdpi.com/article/10.3390/ijms27219496/s1.
