# Supplementary material for: The NAMPT–MMP-9 Axis Contributes to Chronic Inflammation in Cystic Fibrosis
Source: Int J Mol Sci. 2026 Oct 24;27(21):9496. doi: 10.3390/ijms27219496 (PMC13763493; doi:10.3390/ijms27219496)
Supplement: Supplementary file 1 [file ijms-27-09496-s001.zip › ijms-4559207-supplementary.pdf]

Supplementary Information

Table S1. Clinical details of PwCF.

PwCF	AGE	GENDER	CFTR MUTATION	FEV1%
**17**	59	M	F508del/F508del	28
**19**	62	M	F508del/F508del	63
**21**	41	M	F508del/F508del	51
**25**	36	F	F508del/F508del	78
**31**	32	F	F508del/F508del	59
**32**	32	M	F508del/F508del	85
**35**	35	M	F508del/F508del	77
**42**	61	F	F508del/1001+11C>T (introne 6b), M470V (esone 10)	81
**44**	53	M	F508del/3120+kbdel(8.6kb)	65
**68**	37	M	F508del/N1303K	62
**73**	38	M	F508del/G542X	36
**74**	43	M	F508del/F508del	42
**76**	51	M	F508del/F508del	86
**78**	25	M	F508del/F508del	49
**85**	26	F	F508del/E5858X	34
**87**	58	F	F508del/R334W	77
**106**	32	M	F508del/711+1G>T	69
**127**	43	F	F508del/D1152H	-
**128**	54	M	G542X/D1152H	-
**132**	72	F	1898+1G>A/D1152H	-
**146**	69	F	F508del/711+3A>G	-
**150**	23	M	F508del/2184insA	85
**152**	35	M	F508del/dele 22,23,24	-
**154**	28	F	F508del/W1282X	106
**157**	19	F	F508del/I1005R	89
**158**	20	F	M469V/c.3601-3_3610del	124
**162**	17	F	F508del/3120 G>A	131
**164**	20	F	F508del/W1282X	113
**165**	24	F	F508del/F508del	81
**166**	25	F	F508del/F508del	77
**168**	17	M	F508del/F508del	109
**169**	26	M	F508del/F508del	114
**170**	18	M	F508del/F508del	105
**171**	41	F	F508del/F508del	61
**173**	17	M	F508del/S13R	111
**174**	17	M	F508del/2183AA>G	81

**175**	30	F	F508del/L997F/I1027T	28
**176**	21	F	F508del/2595_2596delTG	96
**177**	47	F	F508del/D1152H	77
**179**	34	M	F508del/D110H	92

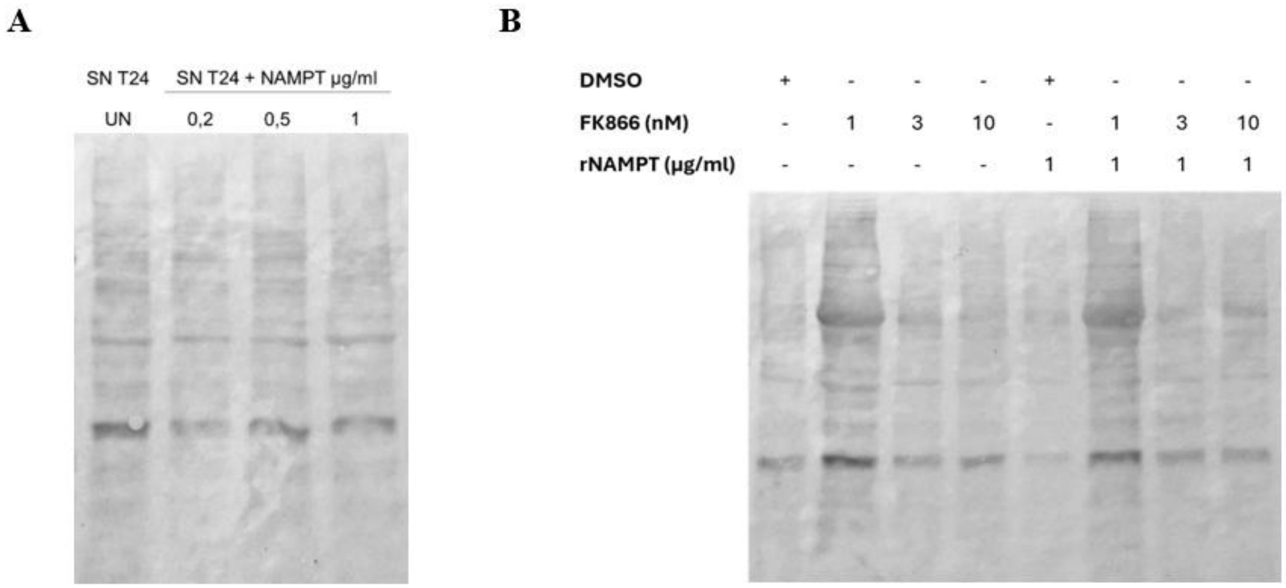

Supplementary Figure 1. Ponceau S staining of the membranes used for total protein normalization.

(A) Membrane corresponding to Figures 3A and 3B. (B) Membrane corresponding to Figure 4A.

FIGURE 1

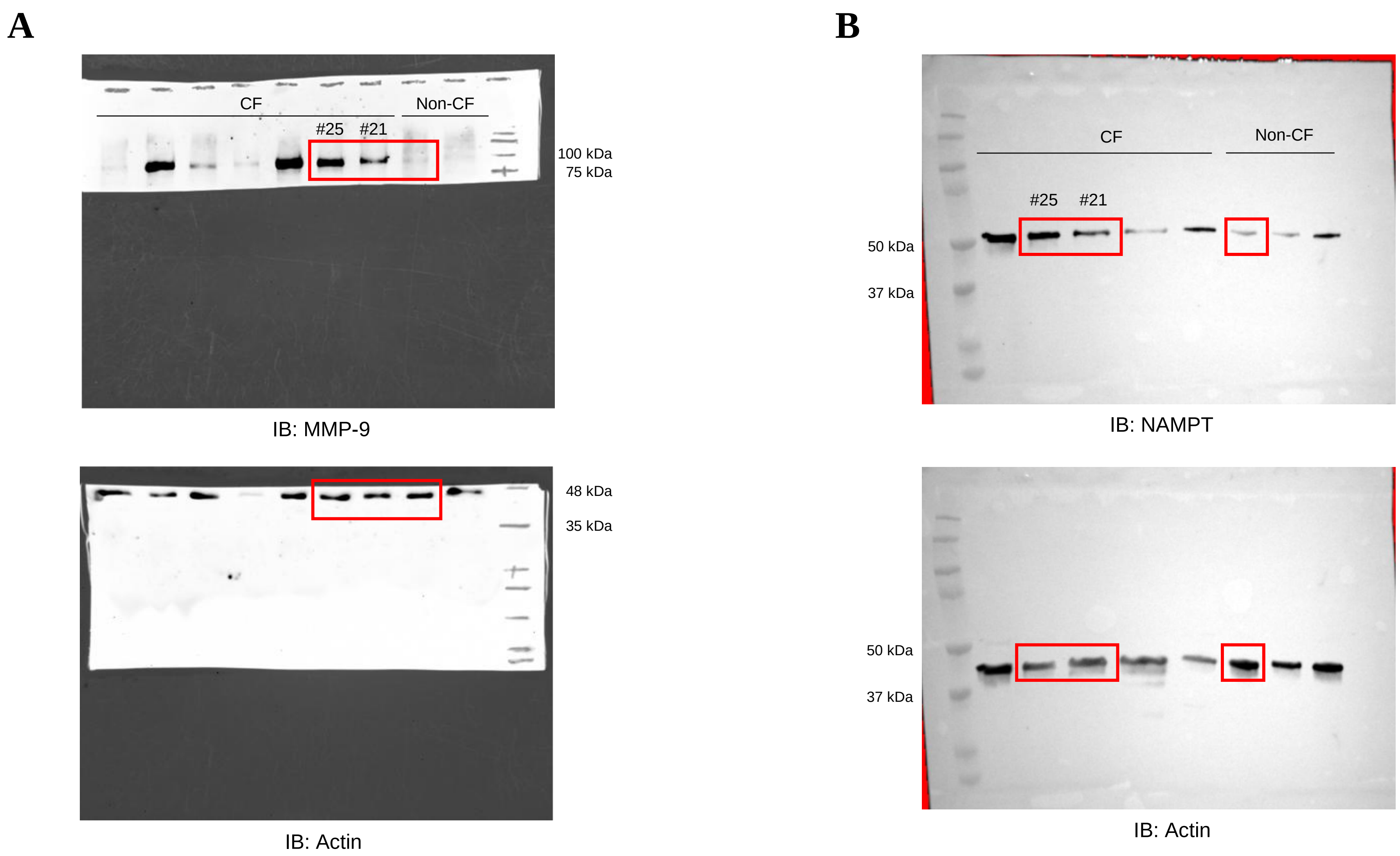
A
CF
Non-CF
#25
#21
100 kDa
75 kDa
IB: MMP-9
48 kDa
35 kDa
IB: Actin
B
CF
Non-CF
#25
#21
50 kDa
37 kDa
IB: NAMPT
50 kDa
37 kDa
IB: Actin

FIGURE 1

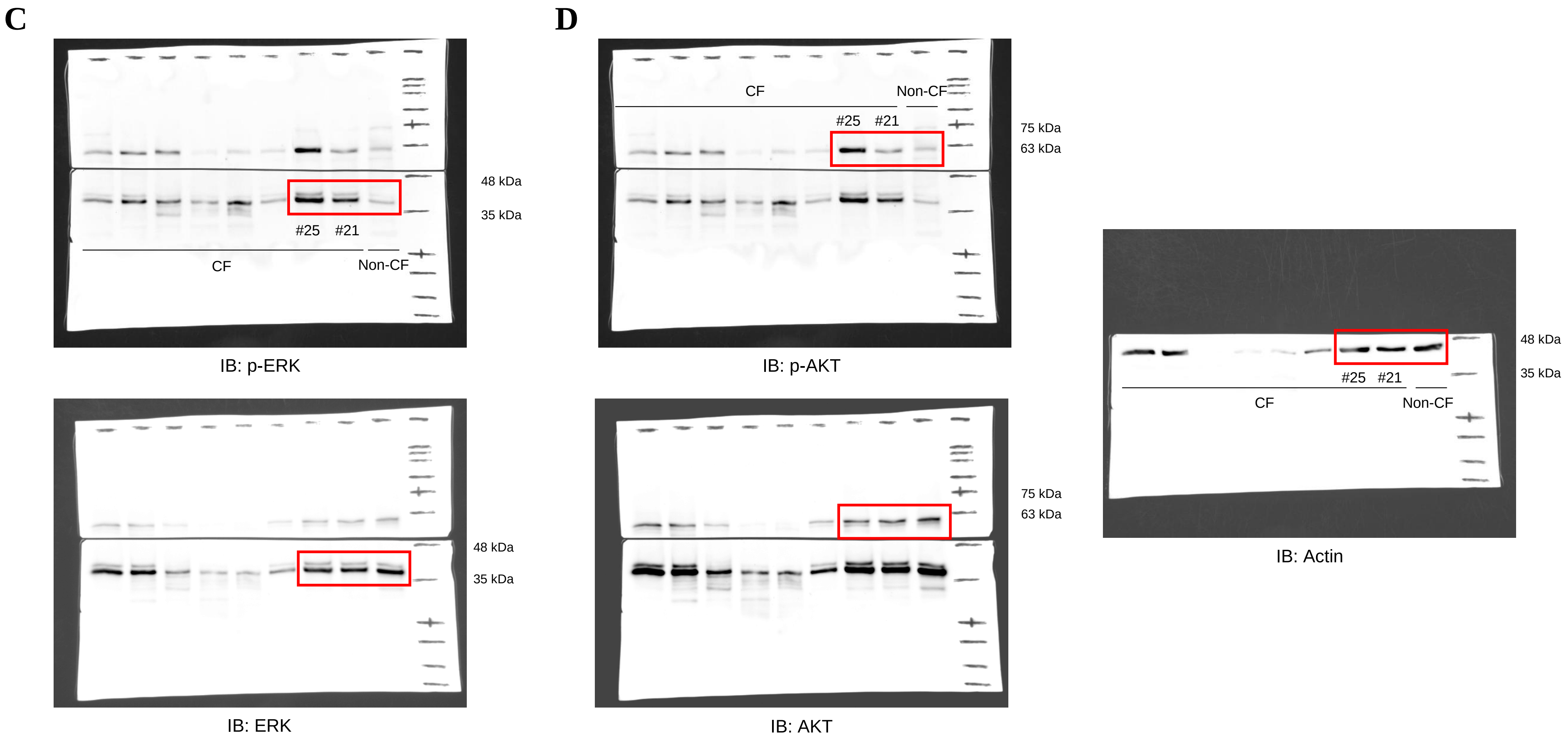
C
D
#25
#21
CF
Non-CF
48 kDa
35 kDa
IB: p-ERK
IB: ERK
75 kDa
63 kDa
IB: p-AKT
IB: AKT
IB: Actin

FIGURE 3

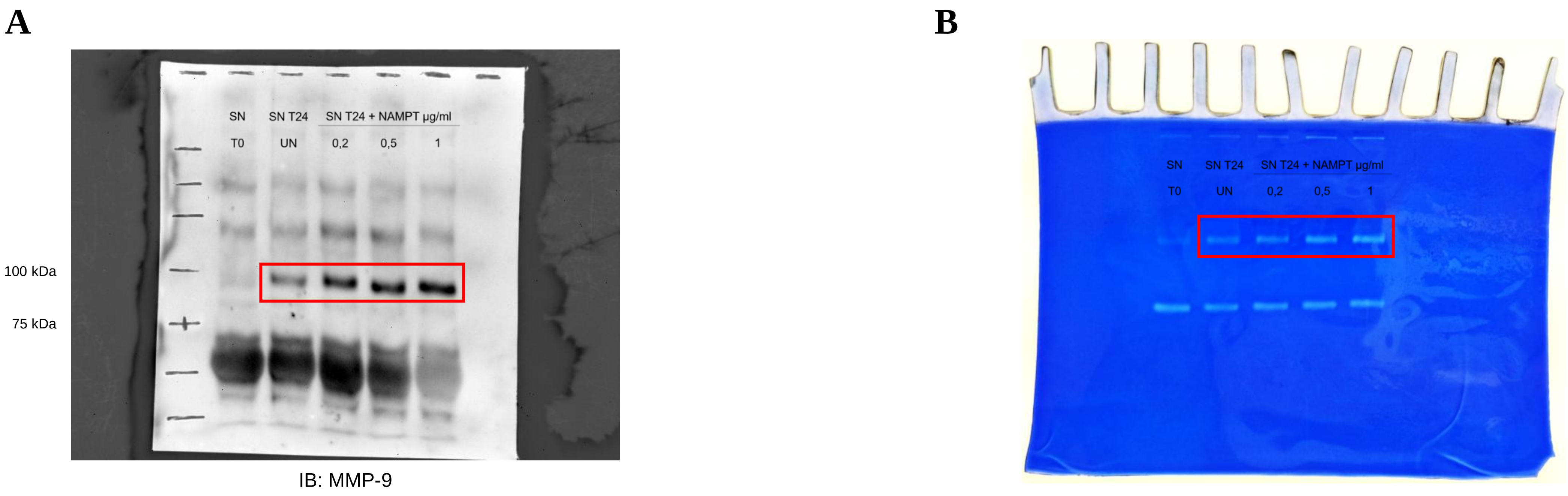

FIGURE 4A

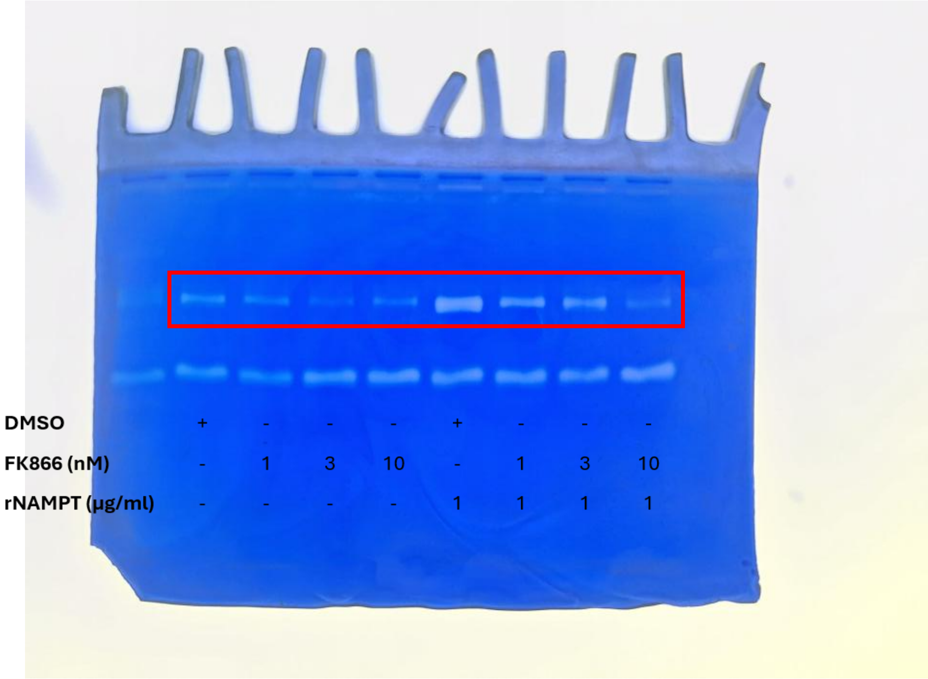

FIGURE 5A

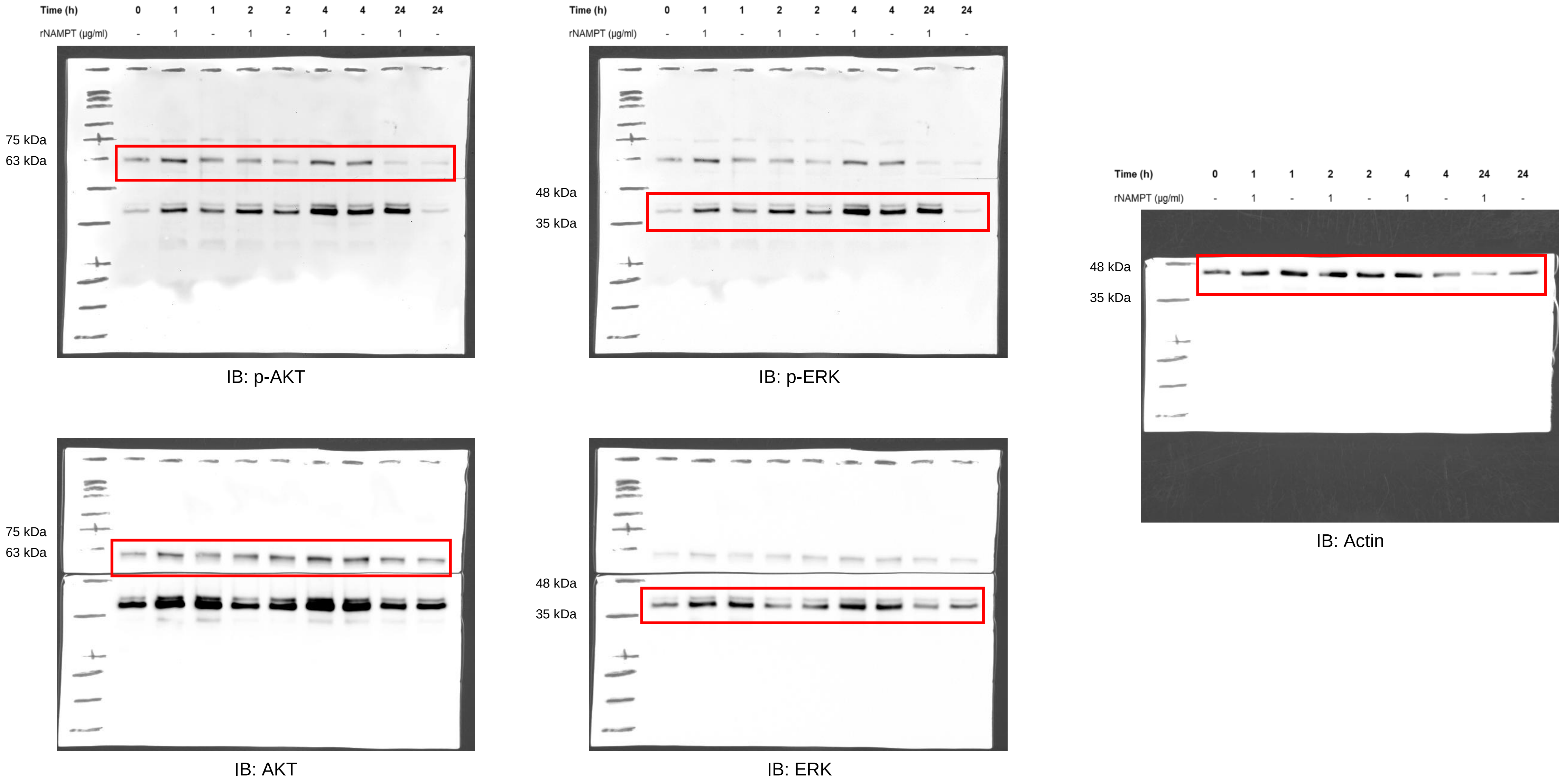
Time (h) 0 1 1 2 2 4 4 24 24
rNAMPT (μg/ml) - 1 - 1 - 1 - 1 -
75 kDa
63 kDa
IB: p-AKT
Time (h) 0 1 1 2 2 4 4 24 24
rNAMPT (μg/ml) - 1 - 1 - 1 - 1 -
48 kDa
35 kDa
IB: p-ERK
Time (h) 0 1 1 2 2 4 4 24 24
rNAMPT (μg/ml) - 1 - 1 - 1 - 1 -
48 kDa
35 kDa
IB: Actin
75 kDa
63 kDa
IB: AKT
48 kDa
35 kDa
IB: ERK

FIGURE 5D

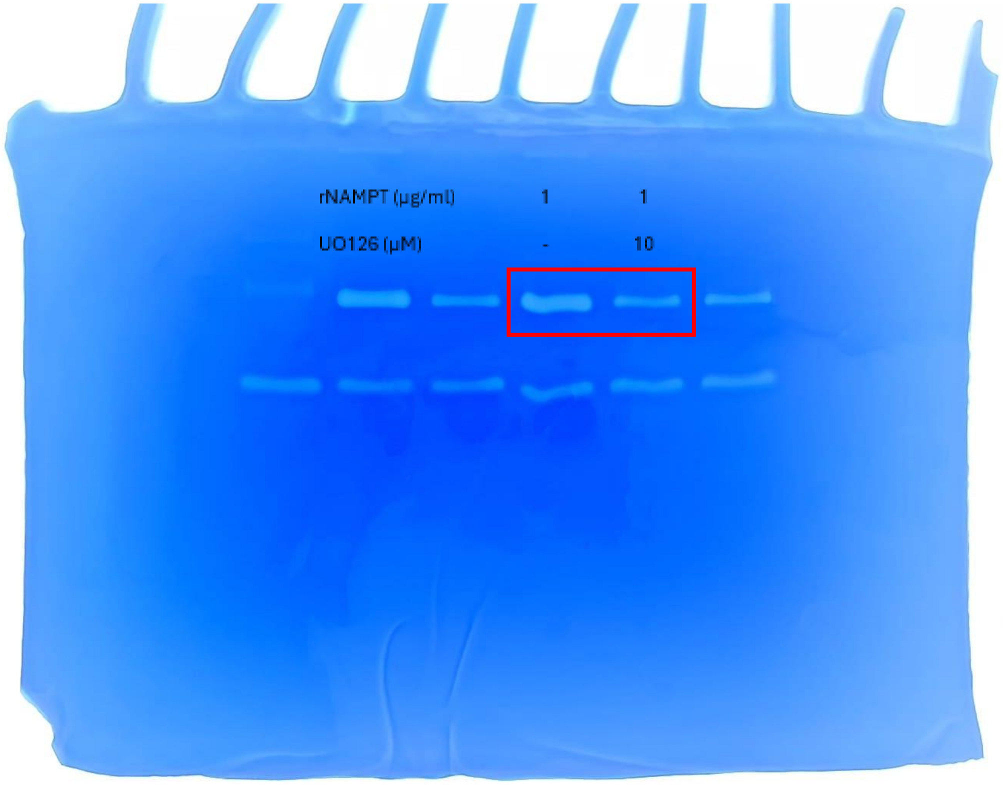

FIGURE 5E

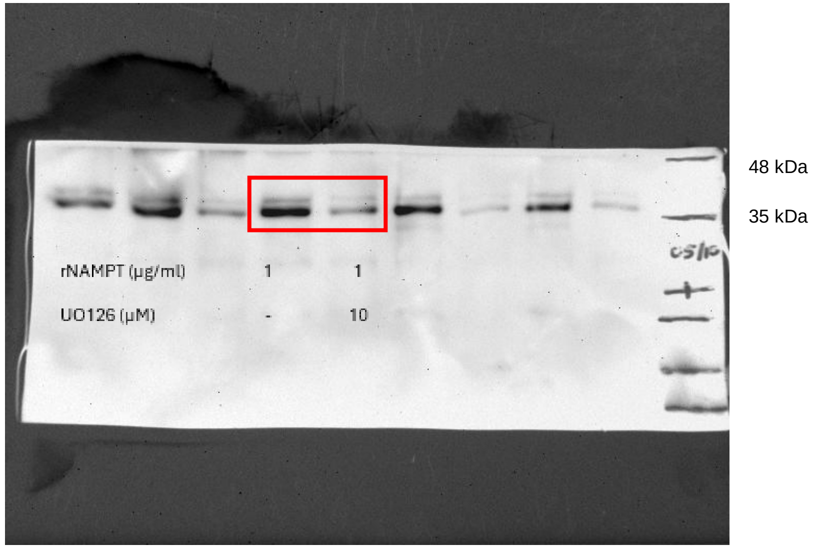

IB: p-ERK

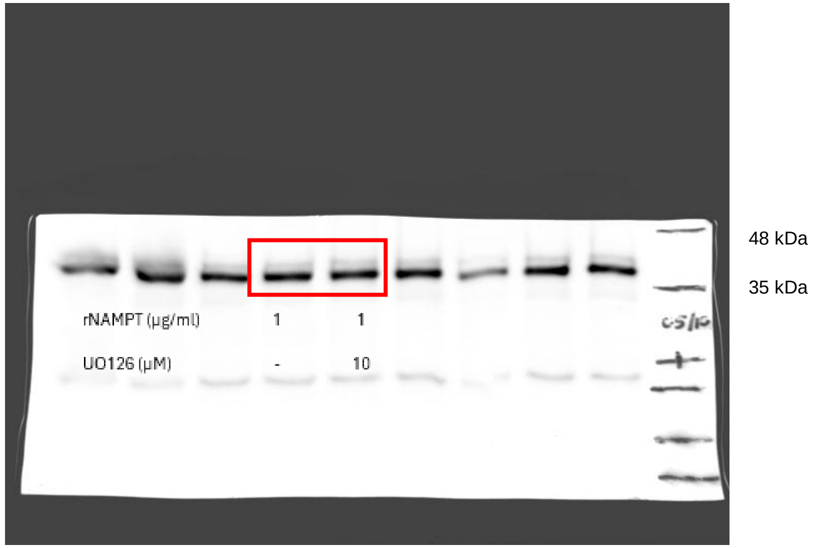

IB: ERK

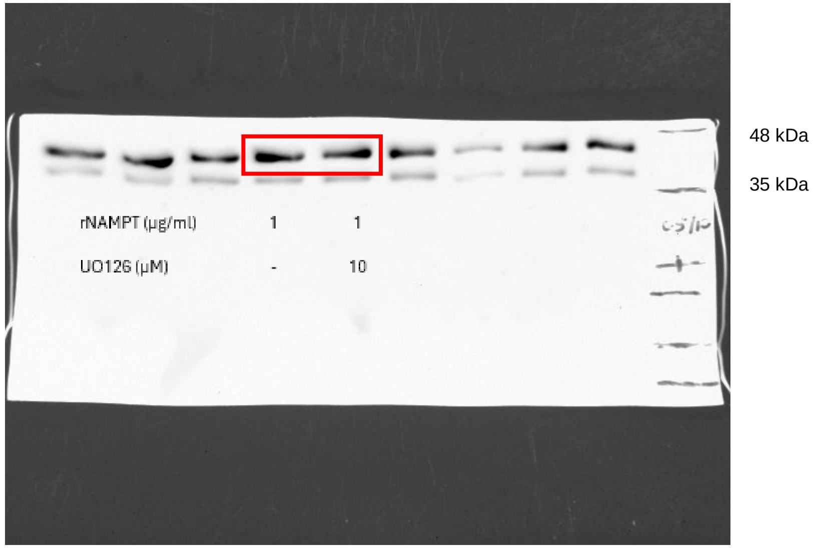

IB: Actin
